# High-Risk Thymoma Hidden Within a Cystic Thymic Lesion: A Diagnostic Clue from Diffusion-Weighted MRI

**DOI:** 10.3390/diagnostics16111680

**Published:** 2026-05-29

**Authors:** Kyungsoo Bae, Hyo Jung An, Kyung Nyeo Jeon

**Affiliations:** 1 Department of Radiology, Institute of Medical Science, School of Medicine, Gyeongsang National University, Jinju 52727, Republic of Korea; ksbae@gnu.ac.kr; 2 Department of Radiology, Gyeongsang National University Changwon Hospital, Changwon 51472, Republic of Korea; 3 Department of Pathology, Busan Paik Hospital, Inje University College of Medicine, Bokji-ro 75, Busan 47392, Republic of Korea; ariel2020@naver.com

**Keywords:** thymoma, thymic cyst, cystic thymoma, diffusion-weighted imaging, apparent diffusion coefficient

## Abstract

Cyst-predominant anterior mediastinal lesions are commonly attributed to benign thymic cysts. However, thymomas—the most common thymic epithelial tumor—may rarely present as a predominantly cystic mass with only a small solid component, posing a diagnostic challenge. The World Health Organization (WHO) classifies thymomas according to the morphology of tumor epithelial cells and the degree of lymphocytic infiltration, a schema that correlates with biological behavior and prognosis. Unlike low-risk subtypes, high-risk thymomas frequently require multimodal treatment because of their more aggressive clinical course. MRI has increasingly been utilized as a problem-solving modality for indeterminate lesions identified on CT. In particular, diffusion-weighted imaging (DWI) with apparent diffusion coefficient (ADC) measurement has emerged as a potentially useful imaging biomarker for predicting histologic subtypes of thymic epithelial tumors. We report a case of a WHO type B3 thymoma presenting as a predominantly cystic anterior mediastinal lesion, in which MRI findings suggested the possibility of a high-risk subtype. The imaging and corresponding histopathologic findings are presented in this paper.

**Figure 1:**
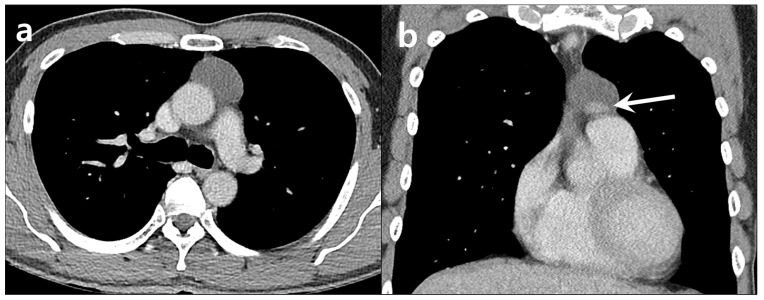
CT images of a 57-year-old man who was referred for surgical resection of an anterior mediastinal mass initially considered to represent a thymic cyst at an outside institution. (**a**) Axial contrast-enhanced CT image demonstrates an approximately 4 cm cystic lesion in the anterior mediastinum with a barely perceptible thin wall. (**b**) Coronal CT image reveals a small oval soft-tissue density (arrow) along the inferior aspect of the cystic lesion, raising concerns for a solid tumor component, an intracystic hemorrhage, or concentrated proteinaceous content [1,2].

**Figure 2:**
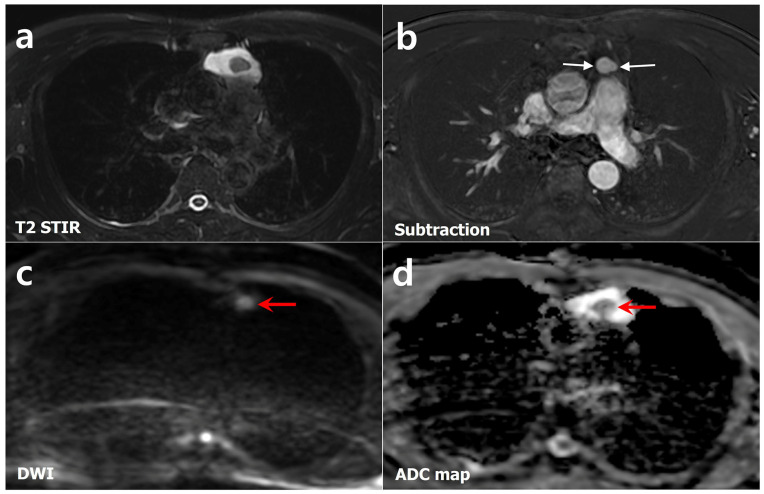
Magnetic resonance imaging (MRI) was performed using a 3-Tesla system. (**a**) Axial T2-weighted STIR image demonstrates a cystic lesion containing a nodular component with intermediate signal intensity. (**b**) Axial subtraction image (post-contrast minus pre-contrast; LAVA-Flex sequence) demonstrates nodular hyperintensity (arrows), confirming true enhancement and supporting the presence of a solid tumor component. (**c**) High b-value diffusion-weighted imaging (DWI; b = 800 s/mm^2^) demonstrates high signal intensity in the nodule (arrow), indicating diffusion restriction, whereas the cystic component appears correspondingly hypointense. (**d**) The apparent diffusion coefficient (ADC) map reveals low signal intensity (arrow) in the nodule. ADC measurement of the nodule using a circular region of interest (ROI), carefully avoiding the cystic area, revealed a low value, with an average of approximately 1.3 × 10^−3^ mm^2^/s from three measurements. Although the present case showed a non-enhancing thin wall and only a very small solid component, the possibility of a high-risk thymoma was suggested based on the MRI findings. The surgeon was informed of this possibility, and preservation of the entire lesion, particularly the solid component, during resection was emphasized to allow accurate histopathologic diagnosis. Through multidisciplinary discussion, the patient was informed preoperatively that adjuvant therapy could be required depending on the final pathological results. Although CT remains the standard imaging modality for anterior mediastinal masses, MRI has increasingly been utilized because of its superior soft tissue contrast and ability to provide functional information [3,4,5,6]. MRI can better depict solid components, thickened septa, and capsules within cystic lesions. In addition, DWI with ADC mapping may provide insights into the cellular density and extracellular space of the lesion. High-risk thymomas and thymic carcinomas tend to demonstrate lower ADC values than low-risk thymomas because of their higher tumor cellularity [7,8,9,10]. Although a cutoff ADC value of 1.309 × 10^−3^ mm^2^/s has been proposed as an optimal threshold for differentiating low-risk from high-risk thymomas [10,11], this cutoff should be applied cautiously in routine practice because thymomas may contain heterogeneous components, such as necrosis, hemorrhage, and calcification. In addition, ADC measurements may be affected by respiratory motion-related misregistration and susceptibility artifacts [11].

**Figure 3:**
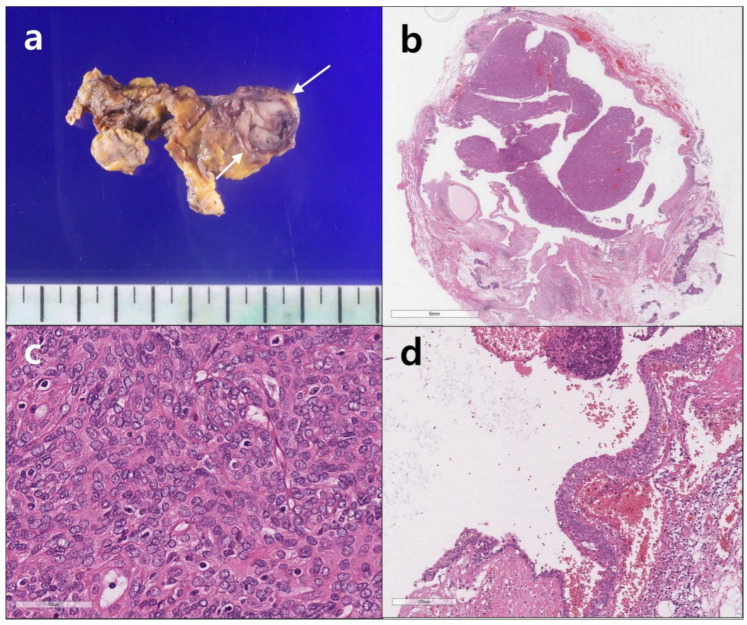
(**a**) Gross specimen of the thymus resected via video-assisted thoracoscopic surgery demonstrates a relatively well-circumscribed, ivory-colored solid mass (arrows) surrounded by a cystic wall. (**b**) Low-magnification view reveals a predominantly solid, hypercellular tumor with an outer cystic component; its cyst wall is lined by a distinct epithelial layer (H&E, ×10). (**c**) High-magnification view demonstrates a predominantly epithelial, hypercellular proliferation composed of relatively monotonous polygonal-to-round tumor cells with sparse mature lymphocytes admixed within the tumor cell sheets, consistent with WHO type B3 thymomas (H&E, ×400). (**d**) The same neoplastic epithelial cells identified in the solid component are also observed along the cyst wall (H&E, ×200). Differing from previous studies reporting cystic thymomas, our case showed a non-enhancing thin wall on imaging studies. Cystic thymomas may arise through two mechanisms: cystic degeneration of thymoma or, less commonly, neoplastic transformation within the wall of a pre-existing thymic cyst. Previous studies have suggested that most cystic thymomas develop through cystic degeneration associated with hemorrhage, necrosis, and dilatation or coalescence of perivascular spaces [9,12,13]. Unlike congenital thymic cysts, which typically show an epithelial lining derived from remnants of the thymopharyngeal duct, cystic degeneration of thymomas usually lacks an epithelial lining and instead demonstrates fibrous and inflammatory cyst walls [9,12]. In the present case, a residual epithelial lining identified along the cyst wall raises the possibility of a thymoma arising from a pre-existing thymic cyst.

**Figure 4:**
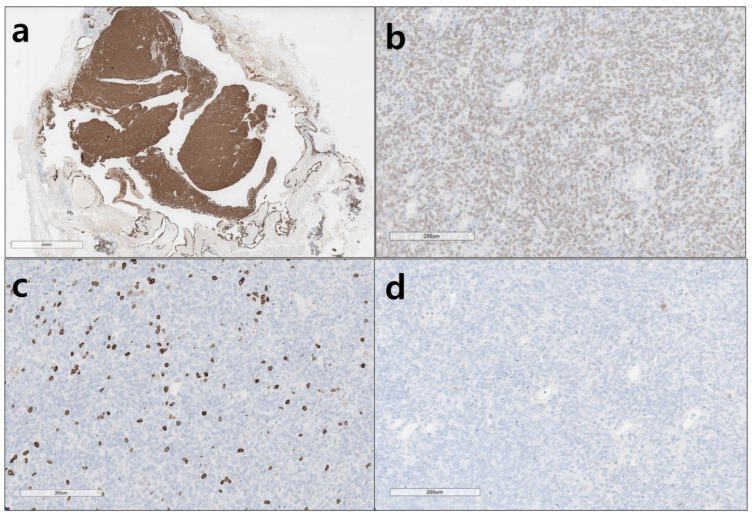
Immunostaining results. (**a**) Cytokeratin (CK) staining demonstrates diffuse positivity in the tumor cells, confirming epithelial differentiation (×10). (**b**) p63 staining shows diffuse nuclear positivity in the neoplastic epithelial cells (×200). (**c**) CD5 staining is negative in the tumor cells, with only focal staining in background reactive lymphocytes (×200). (**d**) c-KIT (CD117) staining is negative in the tumor cells (×200). Diffuse expression of CK and p63 supports thymic epithelial differentiation, whereas the absence of CD5 and c-KIT (CD117) expression argues against thymic carcinoma, thereby confirming the diagnosis of WHO type B3 thymoma. Following surgery, the patient underwent adjuvant radiotherapy to the anterior mediastinum (50.4 Gy in 28 fractions) in accordance with current treatment guidelines and has remained under follow-up without evidence of complications. WHO type B3 thymomas are one of the least common thymoma subtypes, and their presentation as cyst-predominant lesions has been rarely described in the literature [14]. This case highlights the potential additional value of MRI, including DWI and ADC evaluation, in the preoperative characterization of such lesions.

## Data Availability

The raw data supporting the conclusions of this article will be made available by the authors upon request.

## References

[1] Nishino M., Ashiku S.K., Kocher O.N., Thurer R.L., Boiselle P.M., Hatabu H. (2006). The thymus: A comprehensive review. Radiographics.

[2] Koyasu S. (2024). Imaging of thymic epithelial tumors-a clinical practice review. Mediastinum.

[3] Benveniste M.F., Rosado-de-Christenson M.L., Sabloff B.S., Moran C.A., Swisher S.G., Marom E.M. (2011). Role of imaging in the diagnosis, staging, and treatment of thymoma. Radiographics.

[4] Ackman J.B., Wu C.C. (2011). MRI of the thymus. AJR Am. J. Roentgenol..

[5] Strange C.D., Ahuja J., Shroff G.S., Truong M.T., Marom E.M. (2021). Imaging Evaluation of Thymoma and Thymic Carcinoma. Front. Oncol..

[6] Hu Y.C., Wu L., Yan L.F., Wang W., Wang S.M., Chen B.Y., Li G.F., Zhang B., Cui G.B. (2014). Predicting subtypes of thymic epithelial tumors using CT: New perspective based on a comprehensive analysis of 216 patients. Sci. Rep..

[7] Thuy T.T.M., Trang N.T.H., Vy T.T., Duc V.T., Nam N.H., Chien P.C., Nhi L.H.H., Minh L.H.N. (2022). Role of diffusion-weighted MRI in differentiation between benign and malignant anterior mediastinal masses. Front. Oncol..

[8] Abdel Razek A.A., Khairy M., Nada N. (2014). Diffusion-weighted MR imaging in thymic epithelial tumors: Correlation with World Health Organization classification and clinical staging. Radiology.

[9] Yamada D., Matsusako M., Kurihara Y. (2024). Review of clinical and diagnostic imaging of the thymus: From age-related changes to thymic tumors and everything in between. jpn J. Radiol..

[10] Priola A.M., Priola S.M., Giraudo M.T., Gned D., Fornari A., Ferrero B., Ducco L., Veltri A. (2016). Diffusion-weighted magnetic resonance imaging of thymoma: Ability of the Apparent Diffusion Coefficient in predicting the World Health Organization (WHO) classification and the Masaoka-Koga staging system and its prognostic significance on disease-free survival. Eur. Radiol..

[11] Broncano J., Steinbrecher K., Marquis K.M., Raptis C.A., Royuela Del Val J., Vollmer I., Bhalla S., Luna A. (2023). Diffusion-weighted Imaging of the Chest: A Primer for Radiologists. Radiographics.

[12] Suster S., Rosai J. (1992). Cystic thymomas. A clinicopathologic study of ten cases. Cancer.

[13] Sakaguchi Y., Komatsu T., Takubo Y., Terada Y. (2019). Resected case of giant cystic thymoma with spontaneous intracystic hemorrhage. Surg. Case Rep..

[14] Lindholm K.E., Moran C.A. (2019). Cystic and Encapsulated Atypical Thymoma (World Health Organization Type B3). Am. J. Clin. Pathol..

